# Behavioral responses of *Diaphorina citri* to host plant volatiles in multiple-choice olfactometers are affected in interpretable ways by effects of background colors and airflows

**DOI:** 10.1371/journal.pone.0235630

**Published:** 2020-07-06

**Authors:** Haroldo Xavier Linhares Volpe, Odimar Zanuzo Zanardi, Rodrigo Facchini Magnani, Rejane Angélica Grigio Luvizotto, Victoria Esperança, Renato de Freitas, Jennifer Yslaine Delfino, Tatiana Aparecida Mulinari, Rômulo Igor de Carvalho, Nelson Arno Wulff, Marcelo Pedreira de Miranda, Leandro Peña

**Affiliations:** 1 Department of Research and Development, Fund for Citrus Protection (Fundecitrus), Araraquara, São Paulo, Brazil; 2 Chemistry Department, Federal University of São Carlos, São Paulo, Brazil; 3 Instituto de Biología Molecular y Celular de Plantas, Consejo Superior de Investigaciones, Valencia, Spain; US Department of Agriculture, UNITED STATES

## Abstract

In several phytophagous hemipterans, behavior appears to be mediated by both visual and chemical cues. For the Asian citrus psyllid (ACP) *Diaphorina citri* (Hemiptera: Liviidae), olfactometric assays are generally difficult to interpret owing to the low proportion of individuals responding to odors (~30–40%), which compromises the efficiency and reliability of the results of behavioral tests. In the present study, the ACP behavioral response to emitted odors from sweet orange (*Citrus sinensis* L. Osbeck) flushes in a 4-arm olfactometer using different colors (four white-, two white- and two yellow- on opposite sides, or four yellow-colored fields), and the role of the airflow in the concentration of volatile organic compounds (VOCs) were assessed at two airflows [0.4 and 0.1 L/min (LPM)]. Exposure to ‘Pera’ sweet orange or clean air in treatments with four yellow-colored-fields increased the response rate of ACP females to the odor sources compared with exposure to ‘Pera’ sweet orange or clean air in treatments with four white-colored-fields, independently of the odor source and airflow tested. For the assays using two white- and two yellow-colored fields on opposite sides and 0.4 or 0.1 LPM airflow, the residence time of ACP females to odors (‘Pera’ sweet orange or clean air) was similar or higher in treatments using yellow- than those using white-colored fields. For both assays (VOCs and olfactometric behavioral parameters), the reduction in airflow from 0.4 to 0.1 LPM greatly changed the airborne concentration and ACP behavior. Quantitative chemical analyses revelead that the concentration of most compounds emitted by ‘Pera’ sweet orange flushes for the headspace using 0.1 LPM airflow were greater than the concentrations measured using 0.4 LPM airflow. Therefore, this treatment design provides an useful tool to assess the ACP behavioral response to the odors from citrus plants, and it can also help in the discrimination of dose-response screenings for VOCs or conspecific insects.

## Introduction

The Asian citrus psyllid (ACP) *Diaphorina citri* Kuwayama (Hemiptera: Liviidae) is one of the main citrus pests worldwide because it is a vector of the bacteria *Candidatus* Liberibacter spp., which are associated with Huanglongbing, the most devastating disease of citrus worldwide [1]. Among the management methods currently employed, chemical control has been the most successful for suppressing ACP populations and bacterial spread in citrus groves [2]. Although highly efficient, the overuse of insecticides may favor the selection of ACP populations that are resistant to the main insecticide groups [3], pest secondary outbreaks, target-pest resurgence, and beneficial insect mortality, which reduces the effectiveness of the method and the environmental sustainability of the system [4]. Therefore, the development of alternative management tools using volatile organic compounds (VOCs) with attractant [5,6] and repellent [7] properties as lures has gained attention in recent years. However, a thorough understanding of the ecology and cognitive abilities of the pest regarding the finding of both co-specifics and host plants is required [8].

In general, insects use the volatiles emitted by plants/co-specifics to guide them to the odor source [9]. However, long-distance attraction flight studies on sternorrhynchan hemipterans revealed that host plant selection appears to be firstly mediated by visual cues [10–12]. Mound [13] reported that *Bemisia tabaci* (Gennadius) (Hemiptera: Aleyrodidae) is attracted to yellow and blue/ultraviolet (UV) light. Antignus et al. [14] demonstrated that this species positively responded to monochromatic UV light (254–366 nm). For aphids, Döring and Chittka [10] revealed that some species positively responded to colors from the green domain of the spectrum with negative input from the blue and/or UV range. These same authors also suggested that the aphid preference for yellow in field assays was not related to the true color but that additional brightness effects were involved. In terms of long-distance flight for ACP involving only visual cues, Hall et al. [15] demonstrated that yellow stick cards were the most attractive to adults. However, Godfrey et al. [16] showed that both yellow and green stick cards were equally preferred by ACP. Sétamou et al. [17] found that, besides the yellow stick cards, red ones were also effective for catching ACP adults. Miranda et al. [18] reported that ACP take-off and host plant finding ability were disrupted under UV-deficient greenhouse conditions promoted by UV-blocking plastic film coverage. Regarding the emitted light, Paris et al. [19] observed that ACP adults were attracted to UV (390 nm), green (525 nm), and yellow (590 nm) light. Additionally, chemical volatile cues from Rutaceae are important for ACP host-plant location [20–22].

Besides analyzing the influence of emitted light on insect behavior, olfactometric devices are also an important tool for investigating short-distance behavior (by observing insect movement on walking surfaces), which allows for the determination of the influence chemical cues have on insect response. In this sense, Pettersson [23] and Vet et al. [24] developed a 4-arm olfactometer that shows an expressive response to different odors and high foraging activity of hymenopteran parasitoids. This device also simultaneously tests separate VOCs from different odor sources. Besides Hymenoptera, this device has been widely used to assess the behavioral responses of other arthropods to odor sources. However, to our knowledge, no previous study has assessed the influence of 4-arm olfactometer colors and/or airflow in the concentration of emitted volatiles by plants or on the behavioral response of psyllid species. The Y-olfactometer is another short-distance behavior device widely used in behavioral studies of arthropods. This device can be vertically oriented to obtain a high proportion of responding insects for positive phototropic arthropods that tend to move up to the light source. However, the Y-tube configuration induces great turbulence at the junction resulting generally in a mixture of the odors offered [24].

In this context, new short-distance behavior devices and methods were developed to improve the effectiveness of behavioral studies for hemipterans, and particularly for ACP. Wenninger et al. [25] revealed that the addition of emitted light to the Y-tube olfactometer field made source odors more attractive to ACP, indicating that ACP behavior is modulated by both visual and chemical stimuli. Stelinski and Tiwari [26] proposed a new short-distance behavior vertically oriented device named the T-maze olfactometer to study ACP behavior. These authors suggested that the T-maze device was an appropriate tool for assessing hemipteran behavior as they showed positive phototaxis. However, even when ACP does prefer vertically oriented movements, and T-maze or Y-tube olfactometers may be excellent tools to assess attractive odorants [5], they are not appropriate for assessing repellent volatiles as they do not favor an oriented movement of the insect to this kind of odor source, and also because insects could not discriminate some mixed odors. Therefore, these methods are only recommended for attractant odors because repellents also promote an increase in the proportion of non-responding insects [26].

Previous studies have shown a low proportion of responding ACP adults (~30–40%) in assays using repellent compounds in a white 4-arm olfactometer and 0.4 L/min (LPM) airflow [27, 28], hindering data collection. This represents a huge effort for insects, such as ACP, with low foraging activity. Therefore, an olfactometric method for testing repellent volatiles must be developed. Besides the particularities of each assay method, several abiotic factors may influence insect behavior, such as photoperiod, air relative humidity, wind speed, temperature, and barometric pressure [29, 30, 31]. Taking all of these into consideration, there is a limited number of variables that can be calibrated in an olfactometer, such as the color and intensity of the light source, the compostition of the VOCs, the airflow speed to the headspace and the color of the device arms. In terms of airflow, it is known that this variable can disturb whitefly behavior in the olfactometer [32]. Nevertheless, there remains a lack of information on volatiles emitted by plants under the influence of the concentration of airborne compounds when the airflow speed is changed and the effect of that on the behavioral response of psyllids using olfactometers.

Owing to the importance of behavioral studies for the development of alternative tools for suppressing ACP populations and the low proportion of responding psyllids to odor sources in 4-arm olfactometric assays, we hypothesized that the addition of the yellow-colored fields to the device and the reduction in airflow (from 0.4 to 0.1 LPM) in the headspace might increase the proportion of responding ACP adults to the odor sources and alter behavior. This device design constitutes not only a behavioral tool for studying ACP or other insects that present low behavioral responses to odor sources but also for behavioral investigation of insects that use colors as behavioral sources of stimulus. Furthermore, this design will provide higher accuracy and facilitate the feasible screening of dose-response compounds from plants or co-specific insects.

## Materials and methods

### Plants and insects

‘Pera’ sweet orange [*Citrus sinensis* L. (Osbeck)] nursery trees grafted on Rangpur lime [*Citrus limonia* Osbeck, 70–80 cm in height] were grown in 7-L plastic pots with substrate [80% *Pinus* sp. bark, 15% vermiculate, and 5% charcoal (Multplant Citrus^®^; Terra do Paraíso Ltda., Holambra, SP, Brazil)] in a greenhouse. Citrus nursery trees were pruned to 50–60 cm in height at 20 days before the start of the assays to stimulate the production of young shoots. For olfactometric assays, citrus nursery trees with three attached young shoots (10–18 cm in length) were used as odor sources. For behavioral studies, mated ACP females (7–15 days old) from a colony free from *Candidatus* Liberibacter spp. and reared on *Murraya paniculata* (L.) Jack (Rutaceae) seedlings in a climate‐controlled room [temperature 25 ± 2 °C, relative humidity (RH) 65 ± 10%, 14L: 10D h photoperiod, and 3000 lux constant light] were used. The choice of the age of ACP (7–15 days old) was based on Zanardi et al. [5] findings. The authors reported that ACP copulation starts four days after the emergence of adults, with a mating peak at 7 days after emergence. Likewise, Wenninger and Hall [33] observed that opposite ACP sex pairing ≥4 days after emergence yielded 100% mating (females laid only fertile eggs). In this way, the 7–15 days interval ensures that all females were coupled.

The choice of ACP females is due this gender spent more time in citrus odors (grapefruit) field when compared to clean air, while males showed a neutral choice for the same treatment [25], showing that females search for host plant VOCs better than males in olfactometric devices. Mated and single-gender (female) were chosen since we would like to avoid any conspecific volatile that could act as an additional factor to the ACP behavior challenged by citrus VOCs [5, 6]. In the case of mixed ACP behavioral assays, we could wrongly infer that behavioral changes were due to VOCs plants instead conspecific VOCs (e.g. formic, acetic or propionic acids) reported as a male attractant [5, 6] and, in case of acetic acid, found in higher amounts in female extracts [5].

### Behavioral assays

**Fig 1:**
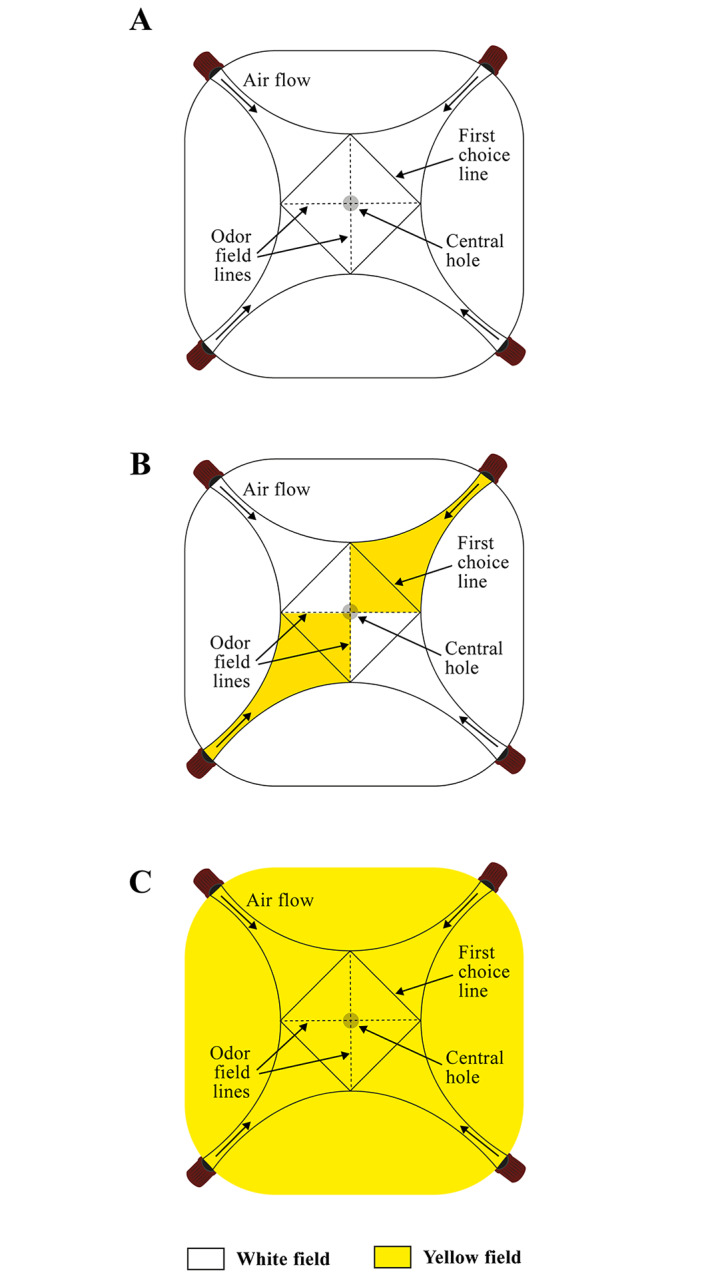
Scheme of 4-arm devices with different colored fields: A) four white-colored fields; B) two white- and two yellow-colored fields on opposite sides; and C) four yellow-colored fields.

*Diaphorina citri* behavioral responses were assessed using a homemade acrylic 4-arm olfactometer (30.0 × 30.0 × 2.5 cm; length × width × height, respectively), with a transparent acrylic lid [24]. In this device, black dotted and continuous lines were marked on the olfactometer lid to allow the insect behavior assessment. In this way, ACP females that do not cross the continuous lines were considered as “non-responding”. Furthermore, four triangular areas (15.4 cm^2^ each) delimited by two dotted lines and one continuous black line marked on the transparent acrylic lid were used to determine the residence time in seconds of ACP females in each odor field. Furthermore, a central hole (1.0 cm in diameter) was made in the center of the olfactometer to allow the odors to leave the device. A 200-μm stainless steel wire braided mesh (2.0 cm in diameter) was placed on the central hole to prevent insects from escaping during the assessment period (Fig 1).

For the present study, the 4-arm olfactometer was modified using four white- (Fig 1A), two white- and two yellow-colored fields on opposite sides of the device (Fig 1B), or four yellow-colored fields (Fig 1C). The yellow-colored fields were made by adding yellow laserjet printed paper below the bottom of the device with the following color spaces: lightness (84.8 ± 0.04), chroma (98.7 ± 0.40), and *hue* angle (95.7 ± 0.02) read with a Minolta CR400 colorimeter (Konica Minolta Co., Osaka, Japan) using visible light (wavelength, 400–700 nm) under room light of 3000 lux. Similarly, the white-colored fields were made by adding blank paper with the following color spaces: lightness (92.6 ± 0.05), chroma (1.3 ± 0.01), and *hue* angle (247.8 ± 0.36). To measure the reflective light from both yellow and white paper, 10 colorimetrical records were used.

For assays, the compressed air used to promote the headspace was produced by a compressed air pump (Schulz MSV6, 120 psi, Planalto Paulista, São Paulo, Brazil) connected to a stainless-steel line with a charcoal filter and humidifier. After being filtered and humified, the clean air was split in four individual 0.635-cm-diameter polytetrafluoroethylene (PTFE) tubes (Sigma-Aldrich, Bellefonte, PA, USA) connected in four flowmeters [0.1–1 LPM, Brooks Instruments, Hatfield, PA, USA], adjusted to 0.1 or 0.4 LPM airflow/flowmeter. Each PTFE tube was connected to one odor source (‘Pera’ sweet orange flushes or clean air), and each airflow converged through PTFE tubes to one of the four device arms.

To provide the plant headspace volatiles, ‘Pera’ sweet orange with three attached young shoots (10–18 cm in length) were used. For this purpose, the young shoots from ‘Pera’ sweet orange nursery trees were enclosed in oven bags cinched with plastic ties at 500 mL volume. Oven bags filled with filtered and humidified air were used as a control (clean air). These bags were not baked since preliminary results showed similar ACP response between non-baked bags filled with clean air × glass chambers filled with clean air.

To understand the role of color and airflow on ACP behavioral response, all possible odor combinations (clean air × clean air, ‘Pera’ sweet orange × clean air, and ‘Pera’ sweet orange × ‘Pera’ sweet orange) were tested in olfactometric assays. For this purpose, the three treatments were assessed using the 4-arm olfactometer with four white-, two white and two yellow-colored fields on opposite sides, or four yellow-colored fields (Fig 1A–1C) using 0.1 or 0.4 LPM airflow (except for the clean air × clean air treatment, which was tested only with 0.4 LPM airflow). Then, a single mated ACP female was released on the central hole of the device and its behavior was observed for 600 seconds after the first choice (across the black continuous lines). For each responding female, the response rate (proportion of responding females concerning total females tested) and residence time (in seconds) in each odor field (triangular area comprising two dotted lines and one continuous black line, Fig 1) were recorded. Females that did not perform a choice (across the black continuous lines, Fig 1) for 300 seconds after their release on the central hole of the device were considered as “non-responding”, and they were not further analyzed. In this way, each responding female was considered as a repetition.

The proportion of responding females and residence time of females in each odor source were adopted as criteria to determine the ACP behavioral response. For each treatment, a dataset of repetitions (responding females) generated on 10 independent assay days (10:00 a.m. to 4:00 p.m.) was analyzed. All assays (behavioral and olfactometric color reflectance) were carried out at 25 ± 1 °C, 65 ± 5% RH, and 3000 lux constants emitted by LED lamps (18 W Philips LED linear tube lights with 4000K and 6500K). The lamps were attached in a double tube stand (containing one 4000K lamp and one 6500K lamp). The double tube stands were evenly distributed in the ceiling to provide an equal amount of light throughout the entire room.

### Volatile emission sampling and analysis by TD-GC-MS

To collect the volatiles emitted by ‘Pera’ sweet orange flushes at 0.1 or 0.4 LPM airflow, 18 or 21 citrus nursery trees with three attached young shoots (10–18 cm in length) were used, respectively. The young shoots were enclosed in oven bags cinched with plastic ties at 500 mL volume. The emitted volatiles were trapped for 30 min at 0.1 or 0.4 LPM airflow in thermal desorption tubes (0.64 cm in diameter × 8.89 cm in length, Supelco) filled with 200 mg Tenax^®^-TA, 35–60 mesh (Sigma-Aldrich, Bellefonte, PA, USA) in a climate-controlled room at 25 ± 1 °C, 65 ± 5% RH, and 3000 lux constant light. A vacuum pump was directly connected to the thermal desorption tubes on the outlet to remove the air from the oven bags using the same airflow used for the inlet (0.1 or 0.4 LPM). Immediately after the volatiles were collected, the young shoots were detached and the fresh mass was weighed using an analytical balance with 0.0001 g precision.

Collected volatiles were thermally desorbed from the tubes in an ULTRA-xr thermal desorption unit with a UNITY-xr automatic sampler (Markes International Ltd., Llantrisant, UK) at 280 °C for 5 min under a helium flow rate of 50 mL/min being concentrated on a cold trap (ref. U-T11GPC-2S, general-purpose carbon; Markes International Ltd., Llantrisant, UK) held at −20 °C. The cold trap was desorbed at 300 °C for 3 min, and the transfer line temperature was set at 200 °C, while the volatiles were released in a splitless mode to a dimethylpolysiloxane capillary column (Rxi^®^-5ms 10 m × 0.10 mm i.d. × 0.10 μm film; Restek Corporation, Bellefonte, PA, USA). The gas chromatography was coupled to a mass spectrometer was a GCMS-QP2010 Plus (Shimadzu Corporation, Kyoto, Japan). The GC temperature program consisted of a start temperature of 40 °C followed by a temperature rate of 20 °C/min to 250 °C, and then held for 2 min. The detector interface and the ion source temperature were 250 and 200 °C, respectively, and the carrier gas was He (50 mL/min, splitless). Mass spectra were recorded at 70 eV, and all analyses were carried out in the total ion chromatogram (TIC) mode with a mass scan range from *m/z* 40 to 450.

Compounds were identified by matching the acquired mass spectra with those stored in reference libraries (NIST and Wiley) and databases from the retention index, related to *n*-alkanes (C_8_–C_20_, Sigma-Aldrich). The relative quantification and emission rate of volatiles emitted by shoots of citrus nursery trees were performed by using 0.25 μg of *β*-caryophyllene (Sigma-Aldrich) as an external standard. The external standard was prepared by connecting the Tenax^®^-TA tubes to a Markes Calibration Solution Loading Rig (CSLR^®^) and spiked with 5-μL aliquots of a *β*-caryophyllene 50 μg mL^–1^ solution under a nitrogen flow of 0.1 LPM. The nitrogen flow was maintained for 5 min after spiking to purge off the methanol used as diluents. The emission rate was expressed in ng g^-1^ fresh weight (FW) L^−1^, where the mass of each volatile compound was quantified in nanograms (relative to the mass of external standard) per gram of FW of the young shoots per liters of air (0.1 LPM = 3.0 L or 0.4 LPM = 12.0 L, considering 30 min of VOCs trapping for both airflows).

### Experimental design and data analysis

All assays were carried out following a fully randomized design. Prior to analysis, the residence time of females in each odor source was converted from seconds to minutes. The proportion data of responding females and residence time of females in each odor field from olfactometric assays and the concentration of VOCs emitted by ‘Pera’ sweet orange flushes were initially submitted to Bartlett´s test using the function “*bartlett*.*test*” to verify the variance homogeneity [34], and the Shapiro-Wilk test using the function ‘‘*shapiro*.*test*” to check the residual normality [35]. Since the proportion data of responding females and residence time of females in each odor field did not meet the assumption of the normal model, the responding female’s data were analyzed through generalized linear models (GLM) [36] using a binomial distribution with a Chi-square test (*P* < 0.05) for separation of means. Meanwhile, the residence time of females in each odor field data was submitted to a non-parametric Wilcoxon matched-pairs signed-rank test (*P* < 0.05) [37]. The concentration of VOC data met the assumption of the normal model and were submitted to ANOVA, and the means were compared by *t*-test (*P* < 0.05). All analyses were performed using the statistical software “*R*” version 3.5.1 [38].

## Results

The four-yellow-colored-field device resulted in a significant increase in the proportion of responding ACP females in relation to the four-white-colored-field device for clean air × clean air (0.4 LPM) (*χ*^*2*^ = 6.8102; *d*.*f*. = 1; *P* = 0.0091), ‘Pera’ sweet orange × clean air (0.4 LPM) (*χ*^*2*^ = 14.805; *d*.*f*. = 1; *P* = 0.0001), ‘Pera’ sweet orange × ‘Pera’ sweet orange (0.4 LPM) (*χ*^*2*^ = 17.143; *d*.*f*. = 1; *P* < 0.0001), ‘Pera’ sweet orange × clean air (0.1 LPM) (*χ*^*2*^ = 42.140; *d*.*f*. = 1; *P* < 0.0001), and ‘Pera’ sweet orange × ‘Pera’ sweet orange (0.1 LPM) (*χ*^*2*^ = 15.619; *d*.*f*. = 1; *P* < 0.0001) treatments (Table 1).

**Table 1:** The Proportion of responding *Diaphorina citri* females to the odor sources using four-white- or four-yellow-colored-field devices and two airflows [0.4 or 0.1 L/min].

Treatment	Airflow (L/min)	Proportion of responding ACP females to the odor sources^1^			
		Four white-colored fields		Four yellow-colored fields	
		N	Responding female	N	Responding female
Clean air × Clean air	0.4	211	0.39 ± 0.03 b	189	0.52 ± 0.04 a
‘Pera’ sweet orange × Clean air	0.4	182	0.45 ± 0.03 b	188	0.64 ± 0.04 a
‘Pera’ sweet orange × ‘Pera’ sweet orange	0.4	215	0.43 ± 0.03 b	193	0.63 ± 0.03 a
‘Pera’ sweet orange × Clean air	0.1	221	0.43 ± 0.03 b	175	0.75 ± 0.03 a
‘Pera’ sweet orange × ‘Pera’ sweet orange	0.1	213	0.42 ± 0.03 b	148	0.63 ± 0.04 a

^1^Data (mean ± SE) followed by the same letter in the same row do not differ significantly by the Chi-square test (*P* < 0.05).

**Fig 2:**
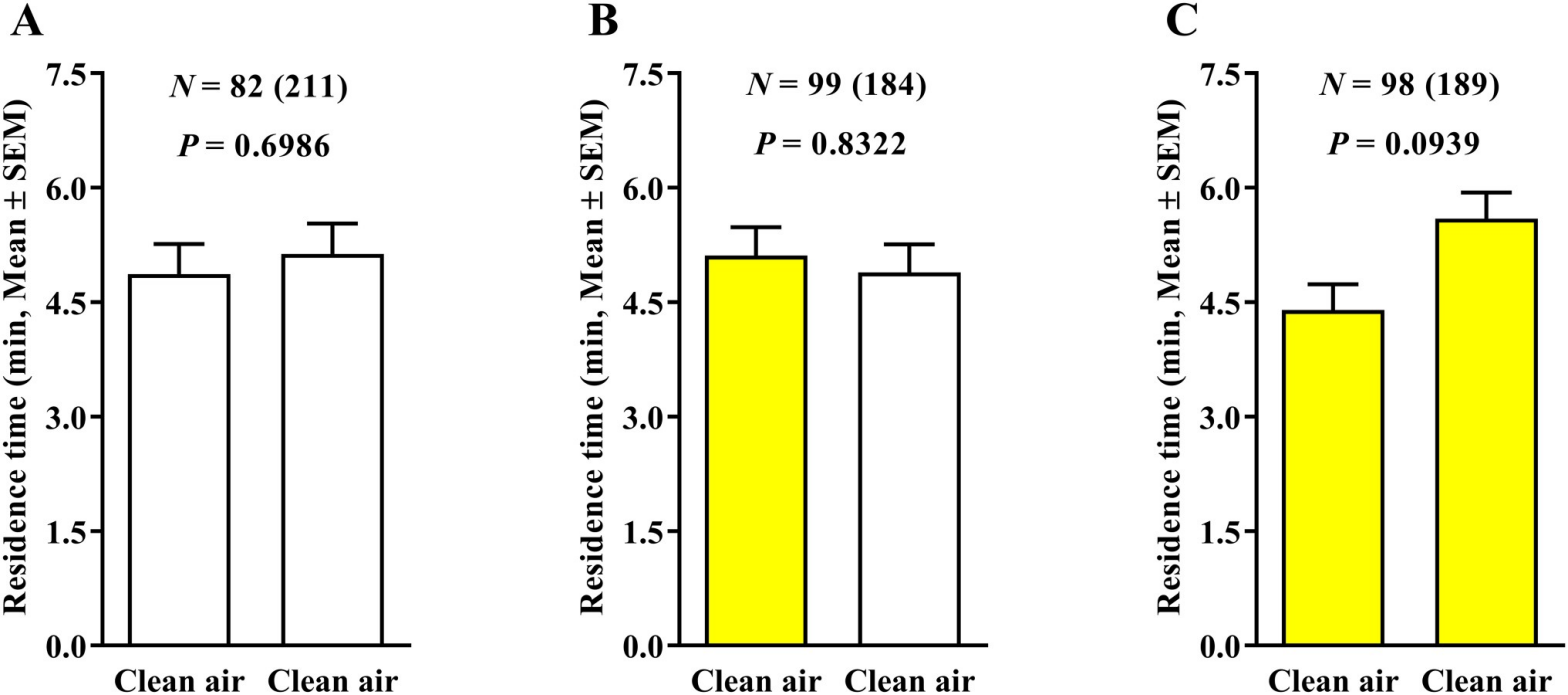
Residence time (in minutes + SE) of *Diaphorina citri* in clean air × clean air treatments (0.4 LPM) using the three 4-arm device color variants. The number of responding psyllids is indicated (N) as is the total number of insects tested (in parentheses) for each treatment. White and yellow bars represent white- and yellow-colored-field devices, respectively. Differences among residence times in the three different color treatments were compared by Wilcoxon signed-rank test (*P* < 0.05).

The olfactometric assays using combinations of white- and yellow-colored fields in three configurations (Fig 1A–1C) showed that, in the absence of ‘Pera’ sweet orange odors (clean air × clean air treatment), no significant difference was observed in the behavior of ACP females released inside the four-white- (Fig 2A), two-white-and-two-yellow- (Fig 2B), or four-yellow-colored-field (Fig 2C) devices. Therefore, the yellow-colored fields did not promote a preference for the field in the clean air × clean air treatment, and neither does the two white- and two yellow-colored fields (Fig 2B).

**Fig 3:**
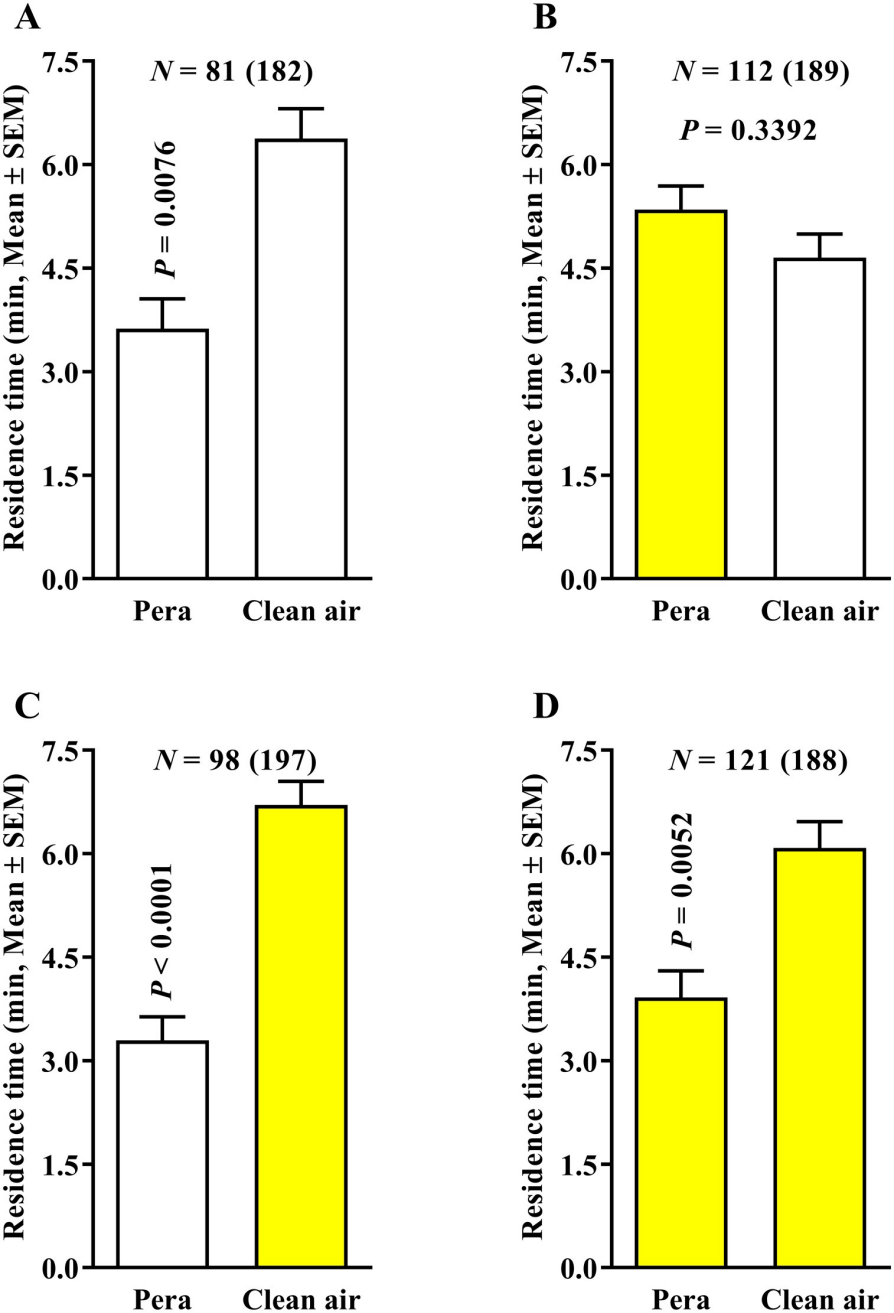
Residence time (in minutes + SE) of *Diaphorina citri* in ‘Pera’ sweet orange × clean air (0.4 LPM) using the three 4-arm device color variants. The number of responding psyllids is indicated (N) as is the total number of insects tested (in parentheses) for each treatment. White and yellow bars represent white- and yellow-colored-field devices, respectively. Differences among residence times in the four different color treatments were compared by Wilcoxon signed-rank test (*P* < 0.05).

When plant volatiles were added to the system (‘Pera’ sweet orange × clean air treatment) under 0.4 LPM airflow, the residence time of ACP females in the fields with clean air was significantly higher than that of those containing ‘Pera’ sweet orange odors for both four white- (Fig 3A) and four yellow-colored fields (Fig 3D), suggesting that such airflow in the olfactometer induced an artifactual response of the psyllids, given that the female would not normally avoid the odor of a host plant. The same behavior was observed in the association of ‘Pera’ sweet orange with two white-colored fields and clean air in the other two yellow-colored fields (Fig 3C). However, in the treatments using two yellow-colored fields with ‘Pera’ sweet orange odors and two white-colored fields with clean air, no significant difference was observed in female ACP behavioral response (Fig 3B).

**Fig 4:**
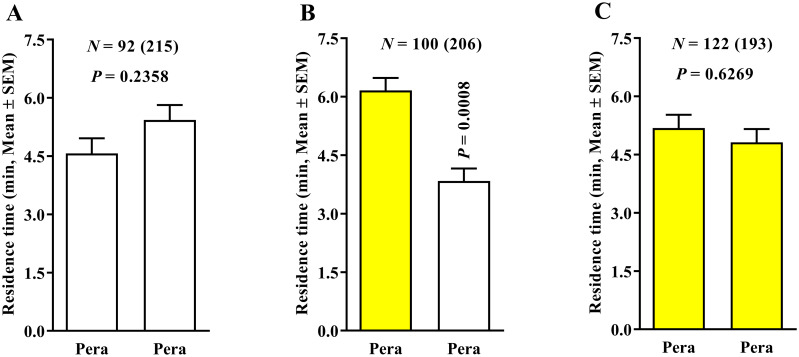
Residence time (in minutes + SE) of *Diaphorina citri* in ‘Pera’ sweet orange × ‘Pera’ sweet orange (0.4 LPM) using three 4-arm device color variants. The number of responding psyllids is indicated (N) as is the total number of insects tested (in parentheses) for each treatment. White and yellow bars represent white- and yellow-colored-field devices, respectively. Differences among residence times in the three different color treatments were compared by Wilcoxon signed-rank test (*P* < 0.05).

In the treatment using ‘Pera’ sweet orange × ‘Pera’ sweet orange and 0.4 LPM airflow, no significant effect was found in the behavioral response of ACP females for both four-white- (Fig 4A) and four-yellow-colored-field (Fig 4C) devices. However, the higher residence time of ACP females was observed in fields using ‘Pera’ sweet orange odors associated with two yellow-colored fields compared with ‘Pera’ sweet orange odors associated with two white-colored fields (Fig 4B). Therefore, in the treatment using the same plant odors and two white- and two yellow-colored fields, the yellow-colored field increased the residence time of ACP females (Fig 4B).

**Fig 5:**
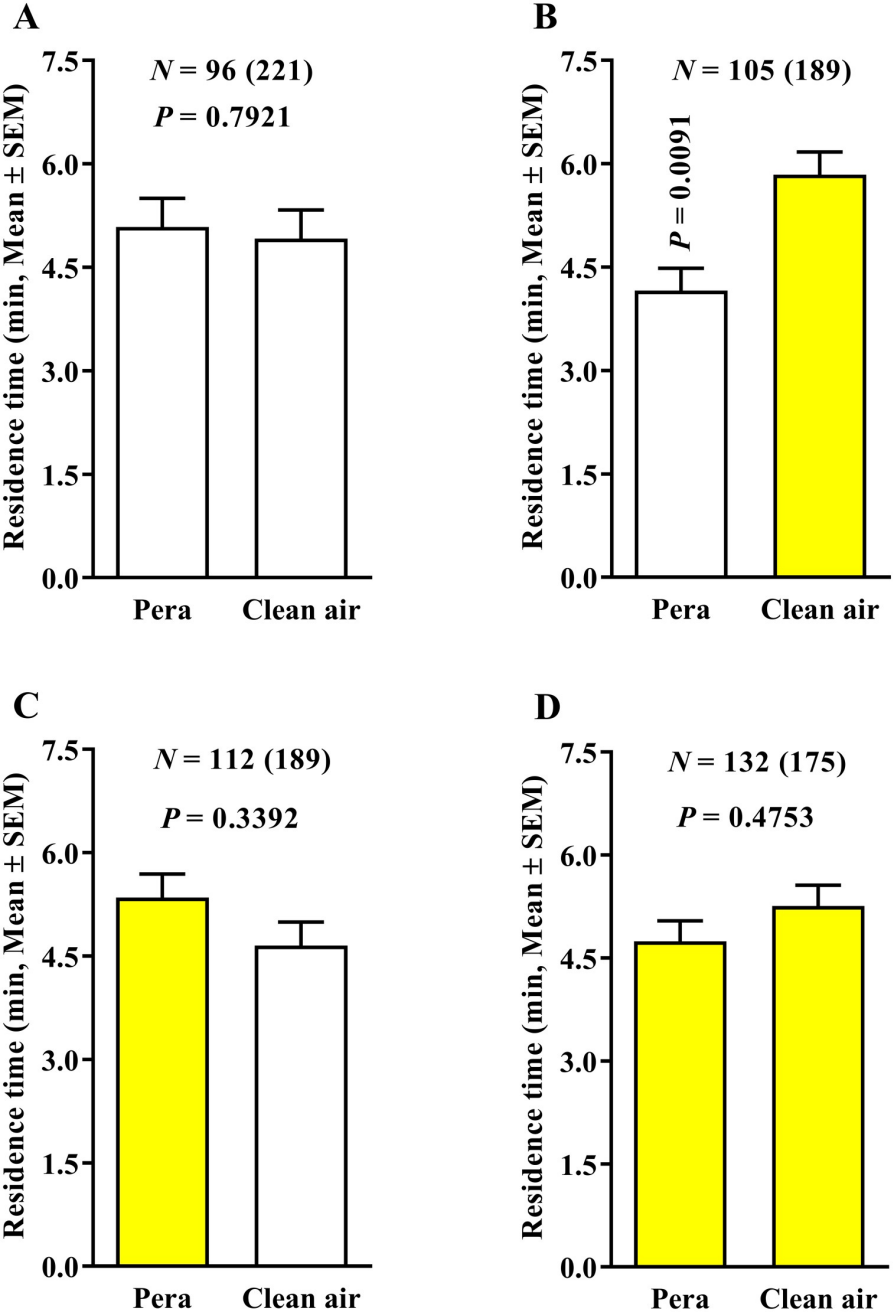
Residence time (in minutes) of *Diaphorina citri* in ‘Pera’ sweet orange × clean air (0.1 LPM) using three 4-arm device color variants. The number of responding psyllids is indicated (N) as is the total number of insects tested (in parentheses) for each treatment. White and yellow bars represent white- and yellow-colored-field devices, respectively. Differences among residence times in the four different color treatments were compared by Wilcoxon signed-rank test (*P* < 0.05).

Next, we tested a reduction in airflow used to avoid the possible artifactual response caused by a high airflow altering the proportions of the ‘Pera’ sweet orange VOC components. The behavior of ACP females was examined in four white-, two white- and two yellow-, and four yellow-colored fields using ‘Pera’ sweet orange × clean air associated with 0.1 LPM airflow to assess the changes in insect behavior. Our results showed that the residence time of ACP females to the ‘Pera’ sweet orange and clean air odors were similar in treatments using four white-colored fields (Fig 5A), yellow-colored fields associated with ‘Pera’ sweet orange odors in two white- and two yellow-colored fields (Fig 5C), and four yellow-colored fields (Fig 5D).

**Fig 6:**
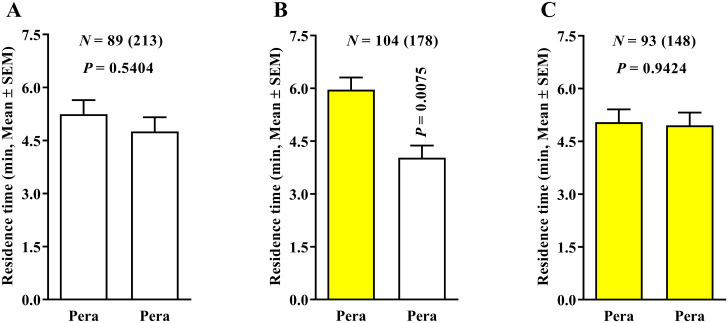
Residence time (in minutes + SE) of *Diaphorina citri* in ‘Pera’ sweet orange × ‘Pera’ sweet orange (0.1 LPM) using three 4-arm device color variants. The number of responding psyllids is indicated (N) as is the total number of insects tested (in parentheses) for each treatment. White and yellow bars represent white- and yellow-colored-field devices, respectively. Differences among residence times in the three different color treatments were compared by Wilcoxon signed-rank test (*P* < 0.05).

Thus, the reduction of airflow from 0.4 to 0.1 LPM changed the ACP behavioral response and provided reliable results. When yellow-colored fields were associated with clean air instead of ‘Pera’ sweet orange in two white- and two yellow-colored fields and 0.1 LPM airflow (Fig 5B), the residence time of ACP females was significantly higher in the yellow-colored fields associated with clean air than those using the yellow-colored fields associated with ‘Pera’ sweet orange odors. Moreover, in the ‘Pera’ sweet orange × ‘Pera’ sweet orange and 0.1 LPM airflow treatment, no significant effect was found in the residence time of ACP females for both four-white- (Fig 6A) and four-yellow-colored-field (Fig 6C) devices. However, a higher residence time was recorded in the treatment using yellow-colored fields associated with ‘Pera’ sweet orange odors instead of white-colored fields with ‘Pera’ sweet orange odors (Fig 6B).

Regarding the behavioral changes in ACP females in the reduction of airflow in the headspace from 0.4 to 0.1 LPM, the volatile rate emitted by ‘Pera’ sweet orange flushes was determined by thermal desorption techniques. The emission rate was expressed as the mass of the compounds emitted by the mass of fresh leaves per liter of air (ng g^−1^ FW L^−1^). Twenty-two VOCs present in the ‘Pera’ sweet orange flushes were identified and quantified comprising 17 monoterpenes and 5 sesquiterpenes (Table 2 and S1 Fig). The predominant volatiles present in the odor of ‘Pera’ sweet orange were the monoterpenes (>90%), comprising the compounds sabinene, limonene, and *trans*-ocimene as the highest emission rates. For the sesquiterpenes, the major compound was (*E*)-*β*-caryophyllene. As shown in Table 2. decreasing the airflow from 0.4 to 0.1 LPM increased the emission rate for all monoterpenes. A similar increase was observed only for the sesquiterpenes (*E*)-*β*-caryophyllene and (*E*,*E*)-*α*-farnesene.

**Table 2:** Identification and emission rate of terpene volatiles from ‘Pera’ sweet orange flushes collected at 0.4 and 0.1 L/min (LPM) airflow in the headspace.

Identification						Emission rate		*P*-value
#	Compound	MF^a^	RT^b^	RI^c^	VP^d^	ng g^–1^ FW L^−1^^e^^,^^f^		
						0.4 LPM	0.1 LPM	
1	*α*-thujene	C_10_H_16_	2.085	930	4.80	0.10 ± 0.01 b	0.44 ± 0.06 a	<0.0001
2	*α*-pinene	C_10_H_16_	2.134	936	4.75	0.22 ± 0.02 b	1.14 ± 0.16 a	<0.0001
3	sabinene	C_10_H_16_	2.433	977	2.60	4.83 ± 0.64 b	20.69 ± 2.96 a	<0.0001
4	*β*-pinene	C_10_H_16_	2.455	980	2.93	0.23 ± 0.03 b	1.15 ± 0.16 a	<0.0001
5	myrcene	C_10_H_16_	2.554	993	2.09	0.25 ± 0.04 b	0.86 ± 0.15 a	0.0008
6	*α*-phellandrene	C_10_H_16_	2.661	1007	1.40	0.03 ± 0.00 b	0.10 ± 0.02 a	0.0007
7	*δ*-3-carene	C_10_H_16_	2.706	1013	1.90	0.25 ± 0.04 b	0.83 ± 0.16 a	0.0020
8	*α*-terpinene	C_10_H_16_	2.756	1019	1.60	0.10 ± 0.02 b	0.41 ± 0.06 a	<0.0001
9	*o*-cymene	C_10_H_16_	2.820	1028	1.50	0.08 ± 0.01 b	0.22 ± 0.02 a	<0.0001
10	limonene	C_10_H_16_	2.852	1032	1.55	0.84 ± 0.17 b	2.72 ± 0.50 a	0.0016
11	*cis*-ocimene	C_10_H_16_	2.913	1040	1.60	0.04 ± 0.00 b	0.10 ± 0.01 a	0.0001
12	*trans*-ocimene	C_10_H_16_	2.997	1050	1.60	1.29 ± 0.21 b	3.69 ± 0.55 a	0.0004
13	*γ*-terpinolene	C_10_H_16_	3.083	1061	1.10	0.20 ± 0.03 b	0.72 ± 0.10 a	<0.0001
14	*cis*-sabinene hydrate	C_10_H_18_O	3.171	1073	0.75	0.03 ± 0.00 b	0.07 ± 0.01 a	0.0072
15	terpinollene	C_10_H_16_	3.317	1091	0.74	0.04 ± 0.01 b	0.15 ± 0.02 a	<0.0001
16	linalool	C_10_H_18_O	3.418	1104	0.16	0.28 ± 0.05 b	0.74 ± 0.15 a	0.0089
17	*α*-terpineol	C_10_H_18_O	4.150	1200	0.04	0.02 ± 0.00 b	0.03 ± 0.01 a	0.0364
18	*β*-elemene	C_15_H_24_	5.581	1398	0.02	0.35 ± 0.05 a	0.42 ± 0.06 a	0.4039
19	(*E*)-*β*-caryophyllene	C_15_H_24_	5.788	1429	0.01	0.48 ± 0.07 b	1.33 ± 0.22 a	0.0012
20	(*E*)-*β*-farnesene	C_15_H_24_	5.999	1460	0.01	0.27 ± 0.06 a	0.29 ± 0.06 a	0.8324
21	*α*-humulene	C_15_H_24_	6.017	1463	0.01	0.14 ± 0.03 a	0.21 ± 0.04 a	0.2235
22	(*E*,*E*)-*α*-farnesene	C_15_H_24_	6.367	1516	0.01	0.10 ± 0.02 b	0.21 ± 0.04 a	0.0154

^a^MF = molecular formula;

^b^RT = retention time (min) on a Rxi-5MS (10 m × 0.10 mm i.d. × 0.10 μm) column;

^c^RI = retention index as determine on a Rxi-5MS column using the homologous series of *n*-hydrocarbons (C_8_–C_20_);

^d^VP = vapor pressure (mmHg at 25°C), source PubChem—open chemistry database at the National Institutes of Health (NIH) (www.pubchem.com) and ChemSpider—open chemistry database at the Royal Society of Chemistry Chemistry (www.chemspider.com);

^e^Expressed as concentration ng g^–1^ FW L^–1^ mean number (0.4 LPM, *n* = 18; 0.1 LPM, *n* = 21) ± SEM from GC-MS data;

^f^Data (mean ± SE) followed by the same letter in a same row do not differ significantly by *t*-test; *P* < 0.05).

## Discussion

In the present study, the increase in the proportion of ACP females responding to the odors in a 4-arm olfactometer by the addition of yellow reflective color and the role of the airflow on the behavioral response of this insect were demonstrated. Our results showed that the four-yellow-colored-field device increased the proportion of ACP females responding to odors compared with the four-white-colored-field device, regardless of the odor source and airflow used in treatments. In the same way, Onagbola et al. [27] reported from 40 to 70% of non-responding ACP in most white 4-arm olfactometer assays when citrus odors associated with some repellents (dimethyl disulfide and/or guava leaves) were tested. These results support the importance of visual cues to hemipterans [10, 11, 12], especially for ACP using colored stick traps [15, 16, 17] and emitted light [19].

However, visual cues alone are not sufficient to significantly induce ACP behavioral response to yellow-colored fields as demonstrated in the treatment using clean air × clean air in a two-white-and-two-yellow-colored-field device. Similarly, Wenninger et al. [25] demonstrated ACP making similar choices to green or white LED light treatments using a Y-tube olfactometer without citrus odors. Thus, assays using ‘Pera’ sweet orange × ‘Pera’ sweet orange in two-white-and-two-yellow-colored-field devices and 0.4 or 0.1 LPM airflow revealed that the yellow-colored fields associated with citrus odors improved the residence time of ACP females on ‘Pera’ sweet orange odor fields. In the same way, an increase in ACP foraging activity was observed in associations of grapefruit odors with green LED lights compared with white LED lights associated with clean air [25]. Patt et al. [39] tested ACP behavioral response to visual, olfactory, and gustatory stimuli, isolated or combined in a Petri dish covered with a black membrane and the center colored with yellow or gray. Afterward, different colored droplets of Specialized Pheromone & Lure Application Technology (SPLAT) were assessed alone or associated with citrus odors. The association of SPLAT colored droplets × middle color did not enhance the response. Therefore, SPLAT droplets associated with citrus odors promoted a higher number of psyllids that entered the device. The results obtained by Wenninger et al. [25] and Patt et al. [39] support our findings that the color associated with odors promotes an increase in the foraging activity of ACP females.

The higher residence time of ACP females in clean air for most of the ‘Pera’ sweet orange × clean air treatments using 0.4 LPM airflow, and after a neutral behavior for the same treatments using 0.1 LPM could be explained by the citrus volatiles emitted by ‘Pera’ sweet orange being diluted at the olfactometer when airflow was increased to 0.4 LPM. Olfactometric assays to determine possible compounds with attractant or repellent properties for insects are concentration dependent. The emitted compound concentration can be altered by diluting the compound source in the releaser (when molecules are tested instead of organisms) or changing the airflow used to perform the headspace. In the case of volatiles emitted by organisms in which compound-emitting rates could not be controlled, as in our assays (e.g., plants, animals, fungi, and bacteria), the only way to change the airborne concentration is to change the airflow speed [40]. These authors reported that increasing the airflow four-fold also decreased the emission rate of compounds four-fold. However, our experiments demonstrated that the increase in all monoterpenes and some sesquiterpenes was not inversely proportional to the reduction of airflow as proposed by Todd et al. [40]. This may be explained by the distinct molecule vapor pressure, ranging from 0.001 to 4.8 mmHg (Table 2), which is intrinsic to each compound. Besides, the proportion of each compound in an odor blend is also fundamental to determine its effects on insect behavior, mainly in complex blends of VOCs emanated by plants. In this way, it has been revealed that insect behavioral response is more associated with a certain proportionality of the blend than with just an individual compound [41, 42].

In the present study, the reduction in airflow from 0.4 to 0.1 LPM increased the concentration (but not composition) of ‘Pera’ sweet orange volatiles in odor fields of the device. Although the techniques used to trap the emitted volatiles from the leaves of sweet orange were different, the volatile compounds identified and quantified in our investigation were similar to those reported in the literature. Killiny and Jones [43] investigated the volatile profile emitted from flushes and mature citrus leaves using a method based on solid-phase microextraction found sabinene, limonene, and ocimene as the main monoterpenes and (*E*)-*β*-caryophyllene as the most abundant sesquiterpenes. However, the residence time of ACP females to ‘Pera’ sweet orange associated with yellow-colored fields was higher than those associated with white-colored fields, regardless of the airflow. These results suggest that, in addition to the odor source, the visual cue is also an important factor to induce significant behavioral responses in ACP females. Wenninger et al. [25] assessed the roles of olfactory and visual cues (yellow-colored sticky card) in the orientation of ACP to four different host plants and found a significant increase in the proportion of responding females and the residence time of females in the arm of the Y-olfactometer releasing volatiles from navel orange or grapefruit associated with yellow-colored sticky card compared with the clean air associated with visual cues.

Our findings suggest that the setup of the olfactometric device plays an important role in ACP behavioral response. Zanardi et al. [5] used a 4-arm olfactometer and verified that ACP males spent more time residing in the acetic acid fields than the control fields. However, for females, the choices and residence time were similar between acetic acid and control fields, suggesting that acetic acid is an ACP male attractant. In another study, Zanardi et al. [6] demonstrated that, besides acetic acid, formic and propionic acids attract virgin ACP males in a 4-arm olfactometer. Tomaseto et al. [44] related an increase of ~30% in ACP preference to orange jasmine volatiles concerning ‘Pera’ sweet orange. All these studies were carried out using the same four yellow-colored olfactometric and 0.1 LPM airflow configuration used herein.

The four-yellow-colored-field device increases the number of responding insects, and consequently, generates more repetition, which increases the accuracy of the olfactometric results. The airflow setup changes the ACP behavioral response, and it is an important factor that should be considered in behavioral studies to understand the activity of chemical compounds for odor-responding insects. The method proposed in the present study, using ACP as a model to understand the role of color and airflow, can be used for investigating other arthropods, especially other hemipterans because these insects normally do not respond as readily in regular 4-arm olfactometric devices, and visual cues play an important role in their behavior. The adoption of the described protocol for other hemipterans can be a step in the study of putative pheromones, kairomones, and allomones. Finally, the standardization of the olfactometric device for the tested species facilitates the comparison of behavioral results from different scientific groups working with the same species.

## Supporting information

S1 Fig Chromatogram of terpene volatiles emitted from new shoots of ‘Pera’ sweet orange nursery trees. (TIFF)
