# Supplementary figures and images for: Behavioral responses of Diaphorina citri to host plant volatiles in multiple-choice olfactometers are affected in interpretable ways by effects of background colors and airflows
Source: PLoS One. 2020 Jul 6;15(7):e0235630. doi: 10.1371/journal.pone.0235630 (PMC7337303; doi:10.1371/journal.pone.0235630)

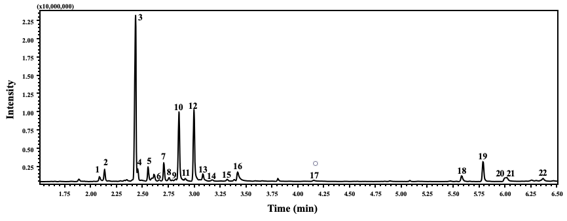

Supplement: S1 Fig — (TIFF) [file pone.0235630.s001.tiff]
